# 3–5 BI-RADs Microcalcifications: Correlation between MRI and Histological Findings

**DOI:** 10.5402/2011/643890

**Published:** 2011-08-25

**Authors:** Valeria Fiaschetti, Chiara Adriana Pistolese, Tommaso Perretta, Elsa Cossu, Chiara Arganini, Claudia Salimbeni, Angela Lia Scarano, Silvia Arduini, Giovanni Simonetti

**Affiliations:** Department of Diagnostic Imaging, Molecular Imaging, Interventional Radiology and Radiotherapy, University of Tor Vergata, Viale Oxford 81, 00133 Rome, Italy

## Abstract

*Purpose*. To evaluate the correlation between MRI and histopathological findings in patients with mammographically detected 3–5 BI-RAD (Breast Imaging Reporting And Data Systems) microcalcifications and to allow a better surgical planning. *Materials and Method*. 62 female Patients (age 50 ± 12) with screening detected 3–5 BI-RAD microcalcifications underwent dynamic 3 T contrast-enhanced breast MRI. After 30-day (range 24–36 days) period, 55 Patients underwent biopsy using stereotactic vacuum-assisted biopsy (VAB), 5 Patients underwent stereotactic mammographically guided biopsy, and 2 Patients underwent MRI-guided VAB. *Results*. Microhistology examination demonstrated 36 malignant lesions and 26 benign lesions. The analysis of MRI findings identified 8 cases of MRI BI-RADS 5, 23 cases of MRI BI-RADS 4, 11 cases of MRI BI-RADS 3, 4 cases type A and 7 cases type B, and 20 cases of MRI BI-RADS 1-2. MRI sensitivity, specificity, positive predictive value, and negative predictive value were 88.8%, 76.9%, 84.2%, and 83.3%, respectively.

## 1. Introduction

Mammographically detected microcalcifications are early diagnosed breast cancers, found in approximately 70% of ductal carcinoma in situ (DCIS) [1, 2]. During the last 20 years, the prevalence of DCIS has grown from less than 5%, before the start of mammographic screening, to 15%–30% in women regularly checked with mammography [3]. The extension of screening mammography has resulted in a decreased number of patients who die of breast cancer, because mammography is sensitive for the detection of clinically occult breast cancer [4, 5]. Mammography has high sensitivity and low specificity, the positive predictive value (PPV) being 15%–30% for malignant nonpalpable lesions [3]. The sensitivity of contrast-enhanced MR imaging in detecting invasive breast cancers has been extensively shown to be very high (94–100%), with a specificity of approximately 65–80% [6]. Conversely, until now, there is no agreement regarding the sensitivity and specificity of breast MRI for the detection of in situ ductal cancers, and the role of MRI in characterizing breast microcalcifications remains a debated issue [7]. In fact the reported values of sensitivity range between 45% and 100% and the specificity between 37% and 95% [8]. A recent study suggested that nonpalpable lesions with microcalcifications categorized as BI-RADS 3 should undergo a biopsy procedure until a more reliable system for the description and classification of microcalcifications is available [9]. VAB has a sufficient sensitivity and specificity to replace surgical biopsy and offers valuable advantages for the evaluation of small concerning lesions and microcalcifications [10].

The purpose of our study was to determine the frequency of malignancy in BI-RADS 3–5 microcalcifications using dynamic contrast-enhanced MR imaging findings in screening detected microcalcification lesions and its correlation with histopathological findings. 

## 2. Materials and Method

### 2.1. Study Population

From January 2007 to December 2009 62 women with BI-RADS 3–5 microcalcifications on mammography underwent breast MRI before a stereotactic biopsy using vacuum-assisted biopsy (VAB). In 8/62 patients the microcalcifications were associated with an opacity, and in 1/62 patients the microcalcifications were associated with glandular distortion. The study received ethics committee approval, and all patients provided informed consent after a clear explanation of the benefits and potential risks.

### 2.2. Mammography

Digital mammographic examinations were performed with GE Senographe DS (General Electric, Milwaukee, USA); mammographic magnification was performed using the view that allowed the best visualisation of the microcalcifications, and the microcalcification extension was evaluated using the standard views.

### 2.3. Mammography Interpretations

The digital mammograms acquired were analyzed in a blinded fashion by two expert radiologists. All cases of microcalcifications were classified according to the method proposed by American College of Radiology [11], and only those classified as BI-RADS 3–5 were selected.

### 2.4. MRI

MR imaging was performed in all cases before microhistology. Dynamic MRI was performed during the 7–14th day of the menstrual period in fertile woman. The instrument was a 3.0 T (Achieva, Philips Healthcare, Best, The Netherlands) MRI apparatus, equipped with 4 channels reception dedicated coil. All the subjects underwent MRI exam with SENSE technology. Patients were examined in prone position with both breasts positioned inside the coil. MRI images were acquired on axial planes. After a survey acquisition, MRI protocol consisted of the following sequences: T1 (TSE) (TR/TE 6.8/3.3 ms; thickness, 3 mm, gap 0; matrix, 512 × 512), T2-TSE (TR/TE 3800/140 ms; thickness, 3 mm, gap 0; matrix, 225 × 512), a short tau inversion recovery sequence (STIR) (TR/TE/TI/4,000/42/155 ms; 3.0 mm, gap 0; 320 × 224), and a T1 dynamic sequence (2D) (FFE) (TR/TE 290/4.6 ms; flip angle, 90°; matrix 256 × 512; thickness, 3 mm; 8 dynamics; with 50 s time resolution for each). The T1 dynamic sequences were acquired by previous 0.1 mmol/Kg gadolinium bolus injection (gadopentetic acid and dimeglumine salt, Magnevist; Schering, Berlin, Germany) administered with a 2 mL/sec flux, followed by a saline flush of 20 mL.

### 2.5. MR Images Analysis

Any contrast enhancement in the area of intermediate microcalcification was considered positive (true positive). The absence of contrast enhancement in the area of intermediate microcalcification was considered negative. All exams were analysed by two radiologists experienced in interpreting breast MRI. During the assessment of MRI exams, the images of the mammography previously performed were available. Each observer assessed the enhancement characteristics, like: shape, nodular (assessment of shape: circular, oval, lobulated, or irregular; edges assessment: smooth, irregular, spiculated) or nonnodular (distribution pattern assessment: nodular, linear/segmental, dendritic). As to the evaluation of the signal/time intensity ratio (*Is*/*t*), regions of interests (ROI) included in the suspect lesions were manually outlined inside the major enhancement areas. The *Is*/*t* curves were characterized depending on the presence of persistent enhancement: type (1) with a continuous increase in signal intensity on each successive contrast-enhanced image; type (2) “plateau pattern” in which an initial increase in signal intensity was followed by a flattening and fluctuation of the enhancement curve; type (3) “washout pattern,” with an initial increase and subsequent decrease in signal intensity. Results of the MRI study were classified into five BIRADS categories: MRI 1-2 negative/benign, MRI 3a probably benign lesion, and MRI 3b borderline lesion/probably malignant; BIRADS 4-5: probably malignant/malignant [11]. The so-called borderline breast lesions may lie on a spectrum of pathological entities which are difficult to distinguish from malignant lesions [2, 3]. The borderline lesions recognised in this study are atypical ductal hyperplasia, flat atypia adenosis, and ductal papilloma. Actually a MRI demonstrated that borderline lesion is referred for a diagnostic surgical biopsy to exclude the presence of a nearly carcinoma lesion.

### 2.6. VAB System

VAB VACORA (BARD) system was adopted to carry out most parts of our biopsies. The system is equipped with a MRI compatible, disposable coaxial stainless steel introducer needed to guide the a-magnetic fine needle up to the area of interest. The needle consists of two stainless steel cannula with 10 Gauge outer diameter; the inner cannula includes a window for the sample collection, connected with the aspiration cylinder. The VACORA BARD system can be employed as handheld device as well; it allows pressure vacuum generation through an electric engine equipped with a microprocessor. One radiologist with 6 year of prior experience in VAB performed the biopsy with the patients prone on a digital stereotactic table. Complete or partial removal of the microcalcifications was assessed in all cases on two-view full-field mammograms immediately. If microcalcifications had been removed completely or almost completely, clips were placed through the 10-gauge probe to identify the VAB site for sequent surgical excision [12, 13]. The histopathological result was correlated with the mammographic findings by both the radiologist and pathologist in all cases.

### 2.7. Histological Diagnosis

The histological findings were classified into two groups, malignant and benign. Malignant lesions included infiltrative ductal cancer, ductal in situ cancer (DCIS), and high-risk lesions as atypical ductal hyperplasia, flat atypia, and ductal papilloma. Lesions not classified as malignant and high-risk lesions were identified as benign lesions.

## 3. Results

Microhistology examination demonstrated 36 malignant lesions and 26 benign lesions. In particular of 36 malignant lesions, 7 were described as infiltrative ductal cancer, 22 as ductal in situ cancer (DCIS), 3 as atypical ductal hyperplasia, 1 as flat atypia, and 3 as ductal papilloma; on the other hand the 26 benignant lesions were represented by 13 apocrine metaplasia, 10 typical ductal hyperplasia and, 3 sclerosing adenosis. The mammographic examination of 62 Patients showed 26 cases of BI-RADS 3, 29 cases of BI-RADS 4, and 7 cases of BI-RADS 5. In 8 patients the microcalcifications were associated with an opacity while in 1 patient the microcalcifications were associated with glandular distortion. The correlation between mammography and histology results demonstrated that of 23 cases of BI-RADS 3, 8 were apocrine metaplasia, 6 were typical ductal hyperplasia, 2 were sclerosing adenosis, 1 were DCIS, 1 was flat atypia, 3 atypical ductal hyperplasia, and 2 ductal papilloma. Of 32 cases of BI-RADS 4, 5 were apocrine metaplasia, 4 typical ductal hyperplasia, 1 was sclerosing adenosis, 20 DCIS, 1 infiltrative ductal cancer, and 1 ductal papilloma; of 7 cases of BI-RADS 5, 1 was DCIS and 6 were infiltrative ductal cancer (Table 1). Mammographic sensitivity, specificity, positive predictive value, and negative predictive value were 80.5%, 61.5%, 74.3%, and 69.5%, respectively. Including only the DCIS findings, we found that mammographic sensitivity, specificity, positive predictive value, and negative predictive value were 95.4%, 61.5%, 67.7%, and 94.1%, respectively. 

**Figure 1:**
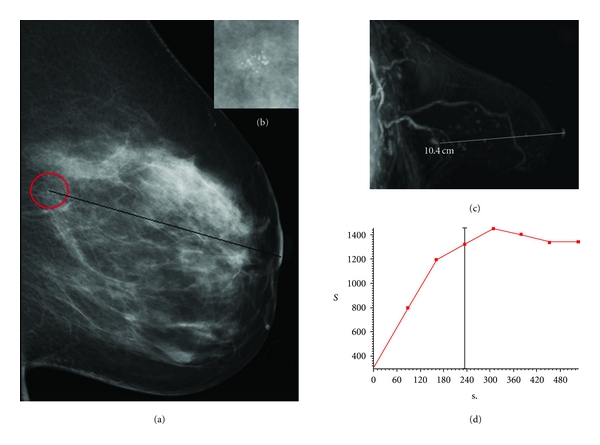
(a-b) A case of MX BI-RADS 4; mammography shows a cluster of microcalcifications in deep upper-outer quadrants of the left breast. (c) MRI shows an irregular area with irregular and segmented contrast-enhancement with longitudinal development (about 15 mm) in deep retroareolar region, next to the cluster of microcalcification seen by Mammography; this case has been classified as MRI BI-RADS 4. (d) time-intensity curve documents heavy but not fast washin and slower washout of contrast-enhancement. Histology demonstrated a DCIS.

**Figure 2:**
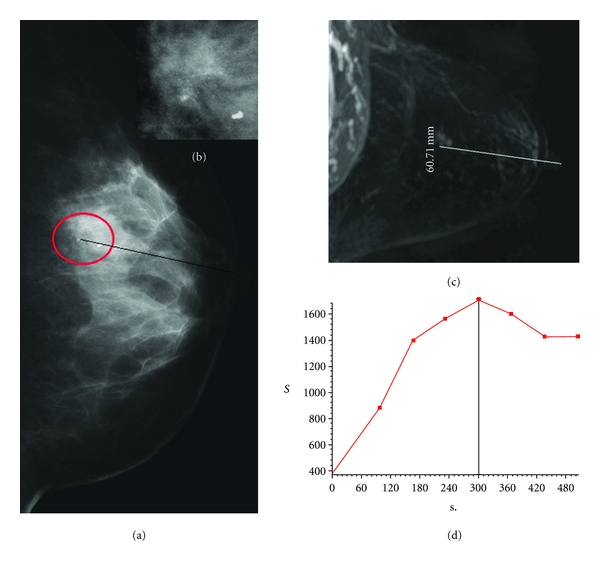
(a-b) A case of MX BI-RADS 3; mammography shows a cluster of microcalcifications in upper quadrants of left breast. (c) MRI shows a millimetric pseudonodular contrast-enhancement without sure substratum in morphologic sequences in the same region of Mx microcalcifications; it has been classified as MRI BI-RADS 4. (d) The time-intensity curve has heavy but not fast washin and subsequent plateau of contrast-enhancement. Histology conversely demonstrated a sclerosing adenosis.

**Figure 3:**
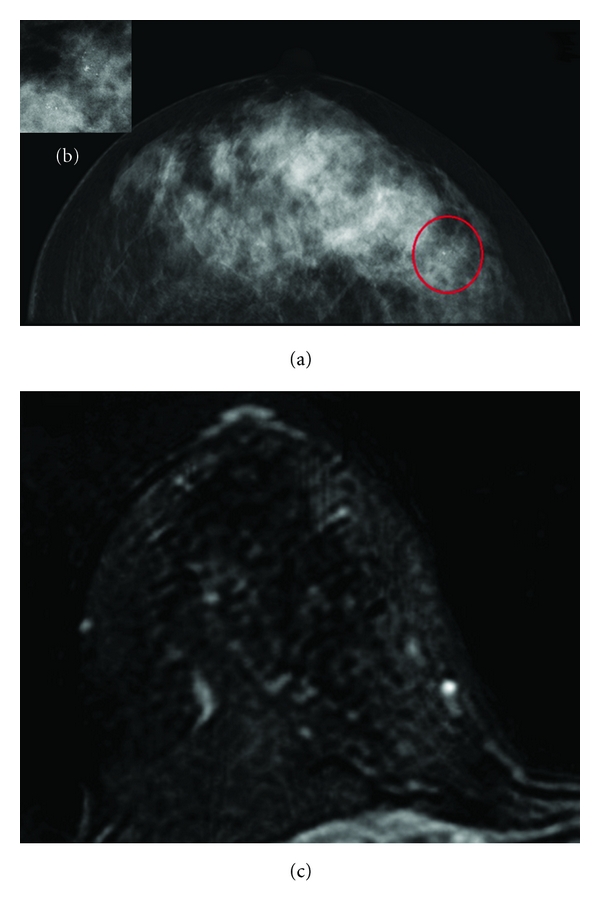
(a-b) A case of MX BI-RADS 4; mammography shows a cluster of microcalcifications in upper-outer quadrants of the right breast. (c) This case has been classified as MRI BI-RADS 1-2 because there was not contrast-enhancement in upper-outer quadrants of the right breast. Histology demonstrated a DCIS.

The analysis of MRI findings identified 8 cases of MRI BI-RADS 5, 23 cases of MRI BI-RADS 4, 11 cases of MRI BI-RADS 3, 4 cases type A and 7 cases type B, and 20 cases of MRI BI-RADS 1-2. The correlation between MRI and histology results showed that of 8 MRI BI-RADS 5, 7 cases were confirmed as infiltrative ductal cancer and 1 as DCIS; of 23 MRI BI-RADS 4, 17 were confirmed by histology as DCIS (Figure 1), whereas 1 were described as apocrine metaplasia, 3 as typical ductal hyperplasia, and 2 as sclerosing adenosis (Figure 2); of MRI BI-RADS 3 type A, 1 was confirmed as apocrine metaplasia, 1 as typical ductal hyperplasia, and 1 as sclerosing adenosis and 1 ductal papilloma; of MRI BI-RADS 3 type B, 2 were confirmed as atypical ductal hyperplasia ad 1 as flat atypia, 2 as DCIS, and 2 ductal papilloma; furthermore of 20 cases of MRI BI-RADS 1-2, 17 were confirmed by histology as 11 apocrine metaplasia and 6 as typical ductal hyperplasia, while 2 were revealed to be DCIS (Figure 3) and 1 to be an atypical ductal hyperplasia (Table 2). MRI sensitivity, specificity, positive predictive value, and negative predictive value were 88.8%, 76.9%, 84.2%, and 83.3%, respectively. Including only the DCIS findings, we found that the MRI sensitivity, specificity, positive predictive value, and negative predictive value were 90.9%, 76.9%, 76.9%, and 90.9%, respectively.

Of 62 patients, 18 (29%) did not show any kind of contrast enhancement at MRI, while 44 (71%) showed contrast enhancement; in particular 7 presented a dendritic enhancement, 18 a linear or segmental enhancement, and 19 a nodular enhancement (Table 3).

On Table 4 we analysed the time intensity curve correlated with the MRI BI-RAD and we showed that of patients with contrast enhancement, 6/44 had a type 1, 16/44 a type 2, and 22/44 a type 3.

Comparing MX and MRI results we found that 2 cases of MX BI-RADS 4, identified subsequently as DCIS, were described as MRI BI-RADS 1-2, because they did not show contrast enhancement (Figure 3).

Despite that, with regard to disease extension, we found that mammography underestimation existed in 6/29 cases (20.9%), confirmed later by histology; in particular of 7 infiltrative ductal cancer histologically detected, MRI permitted to visualise 2 cases of multifocality and 1 case of multicentricity, whereas of 22 DCIS, MRI visualised 2 cases of multifocality and 1 case of multicentricity. 

## 4. Discussion

Mammography is extremely sensitive in detecting microcalcifications even though it does not permit to distinguish malignant from benign lesions and invasive carcinoma from DCIS [14]. In fact in our population sensitivity of mammography in detection of cancer and early cancer related to microcalcifications was 80.5%, and specificity was only 61.5%. The presence of microcalcifications on mammography is often referred to early diagnosed breast cancers and is found in approximately 70% of minimal breast cancers and frequently in DCIS [15]. Stomper and Margolin reports that mammographically detected microcalcifications are the only sign in 72% of clinically occult DCIS lesions [15]. The low specificity of microcalcifications as a feature of malignancy, 10–70% according to literature, is histologically demonstrated in a high number of diagnostic biopsies [14]. On the other hand, approximately 75% of lesions that are detected, suspected, or indeterminate on mammography are revealed to be benign at biopsy [16], suggesting that many biopsies are performed unnecessarily because the indication for VAB has not yet been fully established. 

Dynamic contrast-enhanced MRI is an effective diagnostic technique for symptomatic breast diseases. However its role in evaluating clinically occult disease associated with mammographically detected suspicious microcalcifications has to be clarified. Mammography or ultrasound cannot be replaced by breast MRI even when it is indicated to perform biopsy. Nevertheless contrast-enhanced dynamic breast MRI is actually the best technique in the detection of multifocality or bilateral incidence of carcinoma mainly in dense type of breast. In our study mammography underestimated disease extension in 6 cases (20.9%), in particular of 7 infiltrative ductal cancer histologically detected, MRI permitted to visualise 2 cases of multifocality and 1 case of multicentricity, whereas of 22 DCIS, MRI visualised 2 cases of multifocality and 1 case of multicentricity. Knowing that allowed a better surgical planning in these patients. Previous MR studies have reported variable accuracy of MRI for classification of microcalcifications [17, 18]. Early studies suggested that dynamic contrast-enhanced MRI should not be used to assess microcalcifications [19, 20] because MRI is unable to identify small calcifications, which are typically associated with malignant disease. Westerhof et al. [19] reported a sensitivity of 45% and a specificity of 72% for dynamic MRI in patients with mammographically suspicious microcalcifications. In another study, Gilles et al. [20] observed a sensitivity of 95% and a specificity of only 51% for MRI; in this case, specificity was potentially lower because the presence or absence of contrast uptake in the breast was the only parameter used to define the malignancy. The latest studies have a better rate of diagnosis due to technological improvements. Bazzocchi et al. [17] reported that MRI using up-to-date 3D sequences and combined morphological-kinetic evaluation had a sensitivity of 87%, a specificity of 68%, and an accuracy of 80%. The high variability of MRI diagnostic accuracy in evaluating microcalcifications reported in these studies is related to the use of different criteria as lesion size, histological variability of cancer, cancer angiogenesis, type of enhancement, enhancement pattern, distribution in the breast, margins (regular, irregular), and use of different pulse sequences and scan planes [20]. Cilotti et al. in an analysis of morphological and dynamic features of BIRADS 3–5 microcalcifications observed a sensitivity of MRI of 73%. They considered MRI BIRADS categories 1, 2, and 3 as benign, according to histopathological diagnosis and 4 and 5 as malignant [21]. In our study, the sensitivity of contrast-enhanced MRI was higher (88.8% versus 73% in Cilotti's study). Compared with previous studies, our study could show better results, in addition to the use of up-to-date 3D sequences, probably because of the revised MRI classification system. In fact we proposed a more detailed definition of BIRADS 3 enhancement to detect border-line lesions such as atypical ductal hyperplasia, flat atypia, and ductal papilloma. In our study we observed that in MRI BI-RADS 3 the angiogenesis gave in 8 cases a nodular enhancement and in 3 cases a linear/segmental enhancement. Furthermore we noticed that in 4 cases the time-intensity curve was type 1, in 6 cases was type 2 while in one case was type 3. So correlating the type of enhancement and its time-intensity curve with histological findings, we classified MRI BI-RADS 3 in two subgroups (MRI BI-RADS 3A and MRI BI-RADS 3B). In fact, in 3 cases of MRI BI-RADS 3A the histology revealed 3 benign lesions, and in 5 cases of MRI BI-RADS 3B histology revealed border-line lesions (2 atypical ductal hyperplasia, 1 flat atypia, and 2 ductal papilloma). The so-called border-line breast lesions are lesions which are difficult to distinguish from malignant lesions [22–24]. They are represented by ductal papilloma, atypical ductal hyperplasia, and flat atypia. As defined by the World Health Organization (WHO) Working Group on Pathology and Genetics of Tumors of the Breast [25], epithelial atypia is divided into atypical ductal hyperplasia, flat epithelial atypia (or DIN 1a). Due to a lack of standardized terminology, atypical ductal hyperplasia and flat atypia are sometimes poorly differentiated on pathological examination [26]. As defined by the WHO [23, 24], FEA is an ‘‘intraductal alteration characterized by replacement of the native epithelial cells by a single or 3–5 layers of mildly atypical cells”. However the guide-lines consider to treat the borderline lesions as an early breast cancer and recommend surgical excision. Paying attention to these kind of borderline lesions permits to avoid the transformation of a small lesion into an invasive cancer. In conclusion breast MRI could change the prognosis of the breast cancer detecting the border-line lesions that are recently defined, to be more precise, the precursors of the early breast cancer.

## 5. Conclusions

In conclusion, our study has revealed that contrast-enhanced breast MRI have a better sensitivity and specificity in the evaluation of the early breast cancer related to mammographically detected microcalcifications, than other studies. In fact in our experience breast MRI sensitively improved the percentage of diagnosis of malignancy in mammographically detected microcalcification. This is due to the use of different criteria as lesion size, histological variability of cancer, cancer angiogenesis, type and pattern of enhancement, margins, and use of 3D sequences but specially to a revision of MRI BI-RADS classification, considering the border-line lesions as proposed by the World Health Organization (WHO) Working Group on Pathology and Genetics of Tumors of the Breast.

## Figures and Tables

**Table 1:** Mx BI-RADS and histological correlation.

MX BI-RADS (*N* lesions)	Histology, benign lesions	Histology, malignant lesions
3 (23)	8 apocrine metaplasia	1 DCIS
	6 typical ductal hyperplasia	1 flat atypia
	2 sclerosing adenosis	3 atypical ductal hyperplasia
		2 ductal papilloma

4 (32)	5 apocrine metaplasia	20 DCIS
	4 typical ductal hyperplasia	1 infiltrative ductal cancer
	1 sclerosing adenosis	1 ductal papilloma

5 (7)		1 DCIS
		6 infiltrative ductal cancer

**Table 2:** MRI BI-RADS and histological correlation.

MRI BI-RADS (*N* lesions)	Histology, benign lesions	Histology, malignant lesions
1-2 (20)	11 apocrine metaplasia	2 DCIS
	6 typical ductal hyperplasia	1 atypical ductal hyperplasia

3A (4)	1 apocrine metaplasia	1 ductal papilloma
	1 typical ductal hyperplasia	
	1 sclerosing adenosis	

3B (7)		2 atypical ductal hyperplasia
		1 flat atypia
		2 DCIS
		2 ductal papilloma

4 (23)	1 apocrine metaplasia	17 DCIS
	3 typical ductal hyperplasia	
	2 sclerosing adenosis	

5 (8)		7 infiltrative ductal cancer
		1 DCIS

**Table 3:** MRI enhancement.

MRI BI-RADS	Enhancement			No enhancement
	Dendritc	Linear/segmental	Nodular	
1-2			*2*	*18*
3A			*4*	
3B		*3*	*4*	
4		*14*	*9*	
5	7	*1*		

**Table 4:** MRI time-intensity curve of enhancement.

MRI BI-RADS	Time intensity curve			No enhancement
	Type 1	Type 2	Type 3	
1-2	*2*			*18*
3A	*4*			
3B		*6*	*1*	
4		*10*	*13*	
5			*8*	
